# Supplementary material for: Drug repurposing for aging research using model organisms
Source: Aging Cell. 2017 Jun 16;16(5):1006–15. doi: 10.1111/acel.12626 (PMC5595691; doi:10.1111/acel.12626)
Supplement: Supplementary file 7 — Data S1 Zip‐Archive of all report cards. [file ACEL-16-1006-s007.zip › RC_CLR.pdf]

CLR

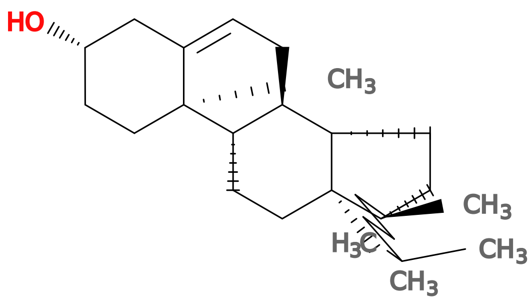

Database identifiers

ChEMBLCompound	CHEMBL112570
CHEBI	16113
ZINC	ZINC03875383
eMolecules	29535203
eMolecules	29536225
eMolecules	482145

Ranking

	Rank	Score
Drosophila	312/697	0.514
C. elegans	426/591	0.123

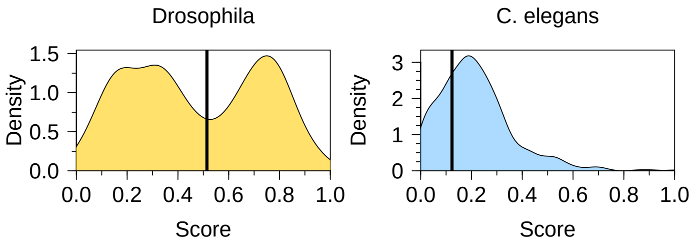

	Ageing implication	Domain conservation	Binding site conservation	Binding affinity	Bioavailability	Lipinski	Promiscuity	Purchasability	Drug approval	**Total**
Drosophila	0.792	0.982	1.0	0.664	(0.9)	-0.05	-0.0	0.1	0.0	0.514
C. elegans	0.792	0.945	0.729	0.664	0.204	-0.05	-0.0	0.1	0.0	0.123

Names

- **cholesterol**
- (3beta,14beta,17alpha)-cholest-5-en-3-ol
- Cholest-5-en-3beta-ol
- Cholestrin

Roles

human metabolite, algal metabolite, mouse metabolite, Daphnia galeata metabolite

Status

Approved drug (according to ChEMBL)	No
Number of Rule of 5 violations	1
Binding affinity to original target in log units (RF-Score prediction)	5.68
Burns *C. elegans* bioavailability prediction	-8.0

Compound Target Characteristics

Transporter

Best gene implication in ageing for this target family came from gene P23977 annotated in UniProt release 2014_02. Annotation GO 7568 (aging) was Inferred from Expression Pattern

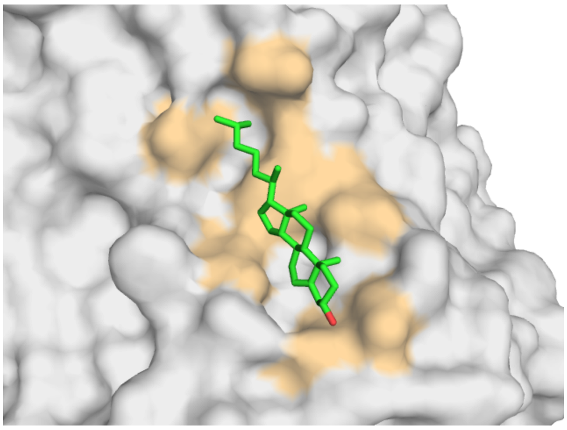

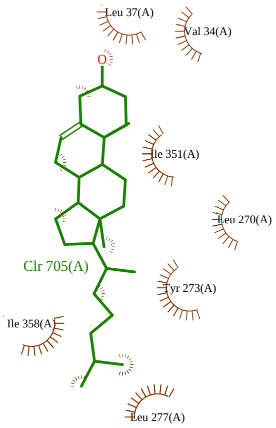

protein	amino acids contacts (binding site)						
PDB:4m48:chainA:Q7K4Y6	V	L	L	Y	L	I	I
sp:Q01959:SC6A3_HUMAN	I	L	T	Y	T	I	S
sp:P23977:SC6A3_RAT	I	L	T	Y	T	I	S
sp:Q61327:SC6A3_MOUSE	I	L	T	Y	T	I	S
tr:Q9NB97:Q9NB97_DROME	V	L	L	Y	L	I	I
tr:Q7K4Y6:Q7K4Y6_DROME	V	L	L	Y	L	I	I
sp:Q03614:NTDO_CAEEL	L	L	L	Y	G	I	L

	whole protein		domain-based		contact-based	
protein	ident	simil	ident	simil	ident	simil
PDB:4m48:chainA:Q7K4Y6	0.99	1.0	0.99	1.0	1.0	1.0
sp:Q01959:SC6A3_HUMAN	0.46	0.75	0.63	0.89	0.43	0.49
sp:P23977:SC6A3_RAT	0.47	0.75	0.65	0.89	0.43	0.49
sp:Q61327:SC6A3_MOUSE	0.47	0.75	0.65	0.89	0.43	0.49
tr:Q9NB97:Q9NB97_DROME	1.0	1.0	1.0	1.0	1.0	1.0
tr:Q7K4Y6:Q7K4Y6_DROME	1.0	1.0	1.0	1.0	1.0	1.0
sp:Q03614:NTDO_CAEEL	0.49	0.77	0.68	0.88	0.57	0.73

DAT (FBgn0034136) associated phenotypes

chemical conditional, circadian rhythm defective, hyperactive, locomotor behavior defective, sleep defective

(Information from FlyBase)

dat-1 (WBGene00000934) associated phenotypes

aqueous adaptation defective, swimming induced paralysis

(Information from WormBase)

dat-1 (UniProt:Q03614) annotation

Function: Terminates the action of dopamine by its high affinity sodium-dependent reuptake into presynaptic terminals. (PubMed:9765501).
Subcellular location: Membrane ; Multi-pass membrane protein
Miscellaneous: This protein is the target of psychomotor stimulants such as amphetamines or cocaine.

(Information from UniProt)
